# The Effect of Argon Plasma Surface Treatment on Poly(lactic-co-glycolic acid)/Collagen-Based Biomaterials for Bone Tissue Engineering

**DOI:** 10.3390/biomimetics7040218

**Published:** 2022-11-29

**Authors:** Phat T. Vu, Jackson P. Conroy, Amy M. Yousefi

**Affiliations:** Department of Chemical, Paper and Biomedical Engineering, College of Engineering and Computing, Miami University, Oxford, OH 45056, USA

**Keywords:** argon plasma, biomimetic composites, poly(lactic-co-glycolic acid), collagen, hydroxyapatite, water contact angle, hydrophilicity, Zisman method, thermal analysis, 3D bioplotting

## Abstract

Nonunion bone fractures can impact the quality of life and represent a major economic burden. Scaffold-based tissue engineering has shown promise as an alternative to bone grafting. Achieving desirable bone reconstruction requires appropriate surface properties, together with optimizing the internal architecture of 3D scaffolds. This study presents the surface modification of poly(lactic-co-glycolic acid) (PLGA), collagen, and PLGA-collagen via an argon plasma treatment. Argon plasma can modify the surface chemistry and topography of biomaterials and improve in vivo integration. Solvent-cast films were prepared using 1,1,1,3,3,3-hexafluoro-2-propanol and characterized via differential scanning calorimetry, thermogravimetric analysis, contact angle measurement, and critical surface tension analysis. For PLGA films, the water contact angle dropped from 70° to 42°, whereas the diiodomethane contact angle reduced from 53° to 32° after the plasma treatment. A set of PLGA-collagen formulations were loaded with nanohydroxyapatite (nHA) and polyethylene glycol (PEG) to enhance their osteoconductivity and hydrophilicity. Then, 3D scaffolds were fabricated using a 3D Bioplotter and characterized via Fourier-transform infrared (FTIR) spectroscopy. A bicinchoninic acid assay (BCA) was used to compare the protein release from the untreated and plasma-treated scaffolds into phosphate-buffered saline (PBS). The plasma-treated scaffolds had a lower protein release, and the difference compared to the untreated scaffolds was statistically significant.

## 1. Introduction

Bone fractures present a serious health burden and negatively impact the quality of life of patients [1,2]. Healthy bone has a remarkable capacity for healing without undergoing scar tissue formation. Nevertheless, bone defects exceeding a critical size (i.e., nonunion) can limit the healing capacity of bone [2]. Bone grafting is currently the “gold standard” treatment for nonunion bone fractures, but it is often associated with some drawbacks such as limited supply, morbidity of the donor site, the risk of failure, and the need to undergo a second surgery [3]. An emerging alternative to bone grafting is scaffold-based tissue engineering, which makes use of a three-dimensional (3D) polymeric matrix that is seeded with cells and implanted in the defect site. The potential for personalized biomimetic designs of 3D scaffolds makes it a promising approach for the treatment of bone defects. Achieving a desirable efficacy for bone reconstruction requires scaffold designs that take into consideration both the bulk and surface properties, together with the internal architecture of the 3D scaffolds [4]. These characteristics can be tuned to facilitate efficient cell adhesion and proliferation throughout the scaffold.

Collagen is a natural polymer derived from animal tissues. Its remarkable cell affinity and low rejection in vivo has made it a desirable material in biomedical research studies [5], and it is commonly used in bone grafting and as a scaffold material for tissue engineering and cell-based gene therapies [6]. The desirability of collagen in bone tissue engineering is due to its abundance, biocompatibility, and low risk of triggering an immune response [7]. Collagen-based scaffolds have been the subject of many research studies due to the auspicious utility of collagen in clinical settings [8]. The cell-binding amino acid sequence (arginine–glycine–aspartic acid) in collagen offers a favorable environment for cell adhesion in vivo. In particular, type I collagen is known for its capability and potential for osteogenic differentiation [9]. Furthermore, collagen is known to be a hemostatic material for wound dressing applications and can promote healing and regeneration [6].

In this study, we prepared formulations containing collagen and poly(lactic-co-glycolic acid) (PLGA)—a biodegradable polymer—owing to its desirable physicochemical and biocompatible characteristics [10]. PLGA is commercially available in various molecular weights and compositions (e.g., lactide/glycolide ratios). It hydrolyzes into lactic acid and glycolic acid, and these products can be safely metabolized inside the body. However, the low hydrophilicity of PLGA leads to its inability to facilitate cell adhesion. Previous studies [9,10] have reported the feasibility of using a gas plasma treatment to improve the surface wettability of PLGA and poly(lactic acid) (PLA) films. These studies have indicated that the gas plasma treatment of polymer surfaces could lead to a significant decrease in their water contact angle [11,12], which was a manifestation of the enhanced cell binding affinity of these biomaterials.

This study focuses on the surface modification of solvent-cast PLGA, collagen, and PLGA-collagen films via an argon plasma treatment. The principle for this modification is plasma ablation, during which the mechanical removal of surface contaminants is stimulated by energetic electrons and ion bombardment. Hence, plasma ablation only affects the outermost molecular layers of the material, confined to a depth of a few nanometers [13]. Argon plasma is often used due to its high ablation efficiency [14]. It has the ability to modify the surface chemistry and topography of biomaterials (e.g., polyurethanes) and improve the angiogenesis and tissue integration of subcutaneous implants [15]. Furthermore, a recent study showed the interplay between argon/argon–oxygen plasmas and gelatin-based biomaterials to enhance their biocompatibility towards biological cells for tissue engineering applications [16].

In this study, the characterization of PLGA, collagen, and PLGA-collagen solvent-cast films before and after exposure to argon plasma was undertaken using differential scanning calorimetry (DSC), thermogravimetric analysis (TGA), contact angle measurement, and critical surface tension analysis via the Zisman method. DSC enables the detection of thermodynamic parameters such as the endothermic/exothermic peak areas, phase transition enthalpy, and melting temperature. TGA allows the study of the thermal instability and decomposition of biomaterials in conjunction with oxidative degradation. Surface characterization through sessile drop contact angle measurement can probe the change in hydrophobicity of a biomaterial surface.

The water contact angle measured in this study was defined as the internal angle of a water droplet sitting horizontally on a material surface and the surface itself [17]. The same measurements were also conducted using diiodomethane, and the contact angles formed between this liquid and the material surface were recorded. Following data acquisition, a Zisman plot was constructed to probe the surface energy of the material. The main principle of the Zisman’s method is based on the previous experimental findings indicating that a freely spreading liquid on a given surface has a surface tension lower than or equal to the surface energy of the material upon which it is spreading [18]. At 20 °C, surface tensions of 72.85 mN/m and 50.77 mN/m have been reported for water and diiodomethane, respectively [19].

The collagen-based formulations prepared in this study were meant to serve as biomimetic precursors for 3D scaffolding, particularly in combination with osteoconductive biomaterials. The solvent-cast films enabled us to conveniently delineate the effects of the plasma treatment on the thermal characteristics and surface properties of PLGA alone, collagen alone, and PLGA-collagen before using them in composite 3D scaffolds containing osteoconductive materials. Composites made of PLGA, collagen, and hydroxyapatite (HA) have shown great potential as biomimetic materials for bone tissue engineering [20,21]. Previous research has shown that HA is osteoconductive [22,23] and helps proteins and cells attach to the scaffold surface when it is used as a component in composite scaffolds [24]. An apatite coating on the surface of biomaterials results in a bone-like biomimetic skin layer and could be a predictive sign of in vivo bone-bonding ability [25]. In particular, nanohydroxyapatite (nHA) can stimulate osteoblast activity and enhance the production of new bone [26,27]. Hence, in this study, a set of PLGA-collagen formulations were loaded with nHA. Polyethylene glycol (PEG) was also added to the formulation to enhance its hydrophilicity and serve as a potential porogen [28], particularly when a multiscale porous network was desirable [29,30]. These composite formulations are currently being investigated in our lab for future in vivo trials.

The prepared PLGA-collagen-nHA-PEG paste was processed using a 3D Bioplotter, and the fabricated 3D scaffolds were characterized using Fourier-transform infrared (FTIR) spectroscopy. A set of 3D scaffolds were subjected to an argon plasma treatment. Subsequently, a bicinchoninic acid assay (BCA) [31] was used to quantify and compare the protein release from the untreated and plasma-treated scaffolds into a phosphate-buffered saline (PBS) solution. The BCA enabled us to detect the concentration of the washed-off protein when the scaffold was placed in PBS. If the collagen undergoes crosslinking upon plasma treatment, it is more likely to be retained within the scaffold formulation. Hence, the extent of the protein dissolved in the PBS solution could probe plasma-induced crosslinking [32] or at least rule out any pronounced degradation of the protein structure upon exposure to argon plasma.

## 2. Materials and Methods

### 2.1. Materials

Bovine type I collagen powder was generously provided by DSM Biomedical (Exton, PA, USA). Diiodomethane (assay > 99%, containing copper as a stabilizer, CAS number 75-11-6), nanohydroxyapatite (synthetic, <200 nm particle size, CAS number 12167-74-7), poly(ethylene glycol) (PEG powder, melting point = 65 °C, CAS number 25322-68-3), and 1,1,1,3,3,3-hexafluoro-2-propanol (HFP) (assay > 99%, boiling point = 59 °C, CAS number 920-66-1) were purchased from Sigma-Aldrich LLC. (St. Louis, MO, USA). PLGA (Resomer^®^ LG-824-S, lactide/glycolide = 82:18, CAS number 30846-39-0) was purchased from Evonik Industries, A.G. (Essen, Germany).

### 2.2. Sample Preparation

#### 2.2.1. Solvent Casting

A Freezer/Mill 6770 (SPEX^®^ SamplePrep, Metuchen, NJ, USA) filled with liquid nitrogen was used to cryogenically grind the PLGA granules. A precool time of 5 min was used, and the grinding was performed for 10 min inside a stainless-steel grinding mill operating at 10 cycles/s. Then, a set of biomaterials films were prepared by the solvent casting method. As shown in Table 1, the formulations consisted of PLGA alone (samples 1 and 2), collagen alone (samples 3 and 4), or a blend of PLGA and collagen (samples 5 and 6) dissolved in HFP.

Specifically, for PLGA–collagen sample preparation, 0.2 g of collagen was transferred to a beaker, and 1.5 mL of HFP was added to the beaker. The mixture was stirred manually for 90 s. Inside a separate beaker, 0.2 g of ground PLGA was dissolved in 0.34 mL of HFP, and the mixture was manually stirred for 120 s. Both beakers were sealed and kept at room temperature for 24 h under a fume hood. The contents of the beakers were then combined and stirred manually for 5 min, and the beaker was sealed and left under the fume hood for 1 h. Subsequently, the dissolved PLGA-collagen mixture was transferred to a 20 mm diameter Teflon mold, and it was left to dry for 14 days without a lid. The finished product after solvent evaporation was a thin film with a thickness of ~1 mm. For PLGA sample preparation, 0.3 g of ground PLGA was mixed with 0.51 mL of HFP, and the mixture was stirred manually for 120 s. For collagen sample preparation, 0.2 g of collagen was mixed with 1.5 mL of HFP, and the mixture was stirred for 120 s. The following steps in the fabrication process were identical to those of the PLGA–collagen sample. The resulting sample was divided into four equal sections using an aseptic razor blade. Then, some of these samples were subjected to a plasma treatment, as shown in Table 1 and described in Section 2.3. The plasma-treated samples were kept inside a desiccator and were characterized within 10 min after the plasma treatment.

#### 2.2.2. Three-Dimensional Bioplotting

A set of 3D scaffolds were fabricated using a 3D Bioplotter (EnvisionTEC, Gladbeck, NRW, Germany). Briefly, 2.1 g of ground PLGA was mixed with 0.9 g of nHA and 3.6 mL of HFP in a 25 mL beaker for 120 s. In addition, 0.225 g of collagen and 3 mL of HFP were mixed for 90 s in another 25 mL beaker. Both beakers were then sealed and kept under a fume hood for 23 h. Then, 0.42 g of ground PEG (20% of PLGA mass) was added to the PLGA-nHA beaker. Finally, the collagen solution was poured into the beaker, and the solution was mixed for 5 min, followed by sonication at an amplitude of 40 μm for 3 min with a Fisherbrand Model 50 Sonic Dismembrator (Fisher Scientific, Portsmouth, NH, USA). The 3D Bioplotter was calibrated prior to scaffold fabrication. The 3D geometry used for 3D plotting was a 20 mm × 20 mm × 3 mm block partitioned into 300 μm layers, which enabled some overlap between the successive layers to prevent delamination [20]. The 3D Bioplotter was set to a pressure of 1.5 bar, a dispensing speed of 2.4 mm/s, and a temperature of 20 °C. The edge-to-edge distance between the plotted strands was set to 1000 μm. The scaffolds were dried under the fume hood for 14 days. The plasma-treated scaffold samples used for the BCA assay were immersed in PBS immediately after the plasma treatment.

### 2.3. Argon Plasma Surface Modification

Only half of the samples received the treatment, while the other half remained as a control group. This included a set of films prepared by solvent casting as well as the 3D scaffolds fabricated using the 3D Bioplotter. The samples underwent 4 min of argon plasma treatment [33] at 0.1–0.2 mbar and 30 W via the PDC-001 Expanded Plasma Cleaner (Harrick Plasma Inc., Ithaca, NY, USA).

### 2.4. Thermogravimetric Analysis

Thermogravimetric analysis (TGA) measurements were performed using a Q500 device (TA Instruments, DE, USA). Samples of approximately 1.5–2.0 mg were placed inside a TGA pan, and the sample mass loss versus time was analyzed from 20 °C to 700 °C at a heating rate of 10 °C/min. The air and nitrogen inflow rates were set at 40 mL/min and 60 mL/min, respectively, to facilitate the combustion of the samples.

### 2.5. Differential Scanning Calorimetry

Differential scanning calorimetry (DSC) measurements were performed using a Q2000 device (TA Instruments, New Castle, DE, USA). Approximately 1.5 mg of the sample was placed inside a standard aluminum pan and press-sealed with an aluminum lid. The device was operated at heating rates of 5 °C/min for PLGA and PLGA–collagen and 2.5 °C/min for collagen from ~20 °C to 250 °C under a nitrogen flow rate of 50 mL/min. The peak temperature of the thermal transitions (T_peak_) as well as the phase transition enthalpy (ΔH) were analyzed via the TA Universal Analysis software.

### 2.6. Contact Angle and Surface Energy Measurements

Sessile drop experiments were performed using a Theta Lite Optical Tensiometer (Nanoscience Instruments, Inc., Phoenix, AZ, USA) on PLGA films (thickness of ~1 mm) before and after the argon plasma treatment (n = 3). A droplet with a 2 μL volume of either deionized water or diiodomethane was used for each contact angle measurement at room temperature. The Zisman surface energy analysis was performed using OneAttension software. A linear graph of the measured contact angle vs. the liquid surface tension was constructed by the software and extrapolated to a contact angle of θ = 0 to estimate the critical surface tension, which approximated the surface energy of the material [18].

### 2.7. Bicinchoninic Acid Assay

Following the argon plasma treatment, all 3D bioplotted samples were submerged in vials of phosphate-buffered saline (PBS) containing calcium and magnesium (PBS++) for ten days at room temperature. A sample mass of ~75 mg in 0.5 mL of PBS++ was used in these experiments. The 10-day incubation period resulted in the partial dissolution of collagen in the PBS++ solution, which was then analyzed through a bicinchoninic acid assay (BCA) [31,34] to quantify the protein concentrations (n = 3). If the structure of collagen was affected by the argon plasma, the concentration of the protein released into the PBS was expected to differ for the plasma-treated and untreated samples. The main principle of the BCA assay is based on two chemical reactions. The first reaction involves the reduction of cupric ions (Cu^2+^) to cuprous ions (Cu^1+^) by the peptide bonds and by some specific residues in an alkaline environment [31,35]. The second reaction involves the chelation of one Cu^1+^ with two BCA molecules, which leads to the formation of an intense purple complex with a peak absorbance at 562 nm. Once a standard curve of absorbance from varying concentrations of bovine serum albumin (BSA) is generated, the protein concentration inside the solution of interest can be quantified by comparing its absorbance at 562 nm with the standard curve [35].

### 2.8. Scanning Electron Microscopy

The 3D bioplotted scaffolds used for scanning electron microscopy (SEM) imaging contained PLGA, collagen, and nHA. The scaffold samples were sputter-coated with a 20 nm layer of gold in a Denton Desk II sputter unit. The microscopy measurements were then performed using a Zeiss Supra 35 VP SEM at an 8 mm working distance and an electron high-tension voltage of 5 kV [20].

### 2.9. Fourier-Transform Infrared Spectroscopy

Infrared spectra were collected with a Harrick Split-pea ATR microscope interfaced to a Perkin-Elmer Frontier Fourier-transform infrared (FTIR) spectrometer. This accessory employed a silicon internal reflection element (IRE) and the standard deuterium triglycine sulfate (DTGS) detector on the Frontier macro bench. The spectra collected using this device represent the average of 32 individual scans possessing a spectral resolution of 4 cm^−1^. The samples were brought into intimate contact with the IRE using a loading of 0.5 kg [36].

### 2.10. Statistical Analysis

The experimental data are presented as means ± standard deviations (SDs). Student’s *t*-test was performed in Excel, and this analysis was used to verify the significance of the differences between the untreated and plasma-treated groups (*p* < 0.05 was considered statistically significant).

## 3. Results

### 3.1. Prepared Films and Scaffolds

**Figure 1:**
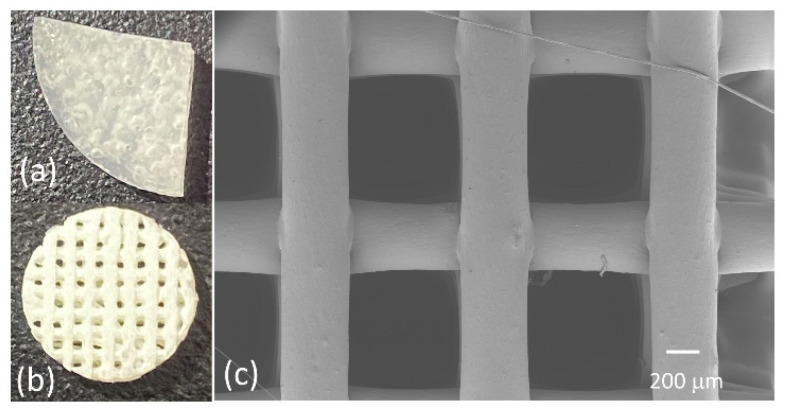
(**a**,**b**) Representative images of the samples fabricated using solvent casting and 3D bioplotting techniques, respectively, (**c**) a top-view image of a 3D bioplotted scaffold, as seen via SEM.

Figure 1a depicts a solvent-cast PLGA-collagen film (sample 5 in Table 1), and Figure 1b shows a 3D bioplotted scaffold. Figure 1c shows a top-view SEM image of a typical 3D bioplotted scaffold made of PLGA-collagen-nHA.

### 3.2. Thermogravimetric Analysis

**Figure 2:**
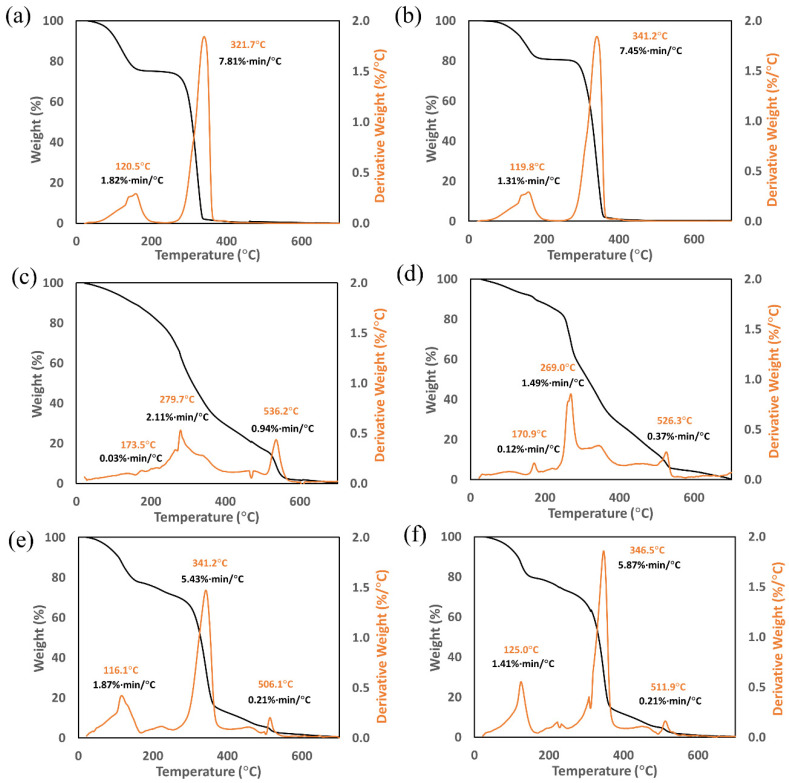
TGA thermograms of the solvent-cast samples prepared using HFP: (**a**) untreated PLGA, (**b**) plasma-treated PLGA, (**c**) untreated collagen, (**d**) plasma-treated collagen, (**e**) untreated PLGA-collagen, and (**f**) plasma-treated PLGA-collagen.

Upon heating at 10 °C/min from 20 °C to 700 °C, the untreated and plasma-treated PLGA samples revealed similar thermal decomposition characteristics regardless of the plasma surface treatment (Figure 2a,b). HFP has a boiling temperature of 59 °C, which marks the onset of sample weight loss in Figure 2a. Despite being a volatile component, the evaporation of the HFP trapped inside the PLGA film appeared to take place at a broad temperature range and peaked at around 120 °C. This was followed by the thermal decomposition of PLGA at a peak temperature range of 322–341 °C (Figure 2a,b). The corresponding TGA thermograms for the untreated and plasma-treated collagen are depicted in Figure 2c,d. The untreated collagen underwent a two-stage thermal event, with an initial degradation peak temperature of 279.7 °C and a secondary degradation temperature of 536.2 °C (Figure 2c). The plasma-treated collagen experienced a similar thermal decomposition, with an initial degradation peak temperature of 269.0 °C and a secondary degradation peak temperature of 526.3 °C (Figure 2d). Finally, as shown in Figure 2e,f, the peak decomposition temperatures for the untreated and plasma-treated PLGA-collagen samples showed the combined characteristics of PLGA (Figure 2a,b) and collagen (Figure 2c,d).

The decomposition of PLGA remained within a narrow temperature range (322–346 °C) whether untreated or plasma-treated and with or without collagen participation in the formulation. In the combined PLGA–collagen samples (Figure 2e,f), the evaporation temperature and maximum mass loss rate of HFP in the collagen samples were countervailed by those in the PLGA samples, resulting in PLGA-skewed parameters for HFP. Overall, the argon plasma treatment appeared to somewhat increase the degradation temperature of PLGA (321.7 °C vs. 341.2 °C, Figure 2a,b) and had a negligible effect on the degradation temperature of PLGA–collagen (341.2 °C vs. 346.5 °C, Figure 2e,f). As for collagen (Figure 2c,d), the secondary degradation peak temperature somewhat decreased for the plasma-treated samples (536.2 °C vs. 526.3 °C), although the area under the peak dropped by over two-fold upon plasma treatment, which potentially made the overall effect negligible. Interpretation of the primary degradation peak temperature in Figure 2c,d is not possible due to the evaporation of the HFP entrapped in the collagen films (See Figure S1, Supplementary Materials).

### 3.3. Differential Scanning Calorimetry

**Figure 3:**
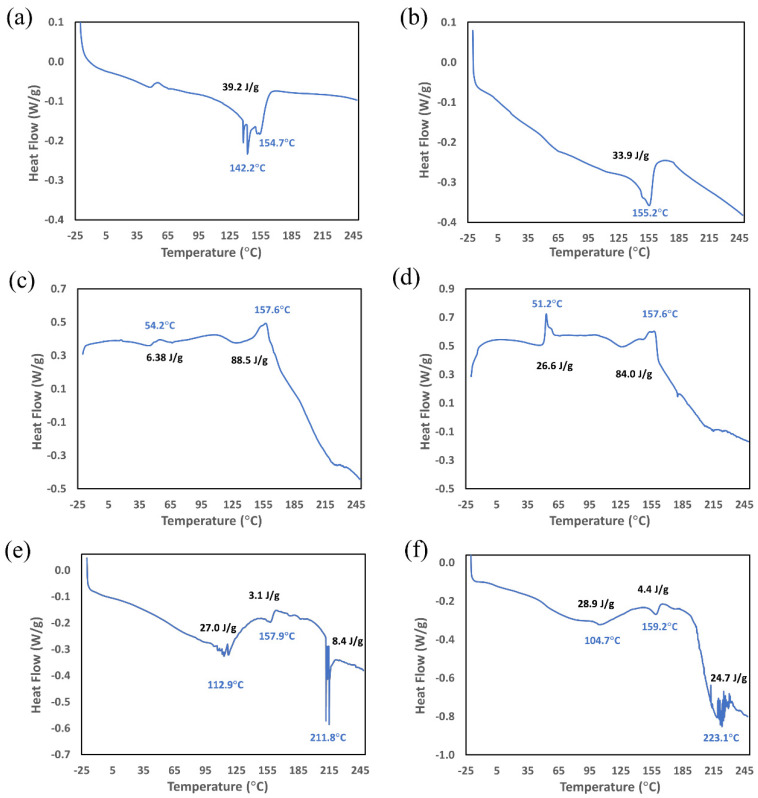
DSC thermogram of the solvent-cast samples prepared using HFP: (**a**) untreated PLGA, (**b**) plasma-treated PLGA, (**c**) untreated collagen, (**d**) plasma-treated collagen, (**e**) untreated PLGA-collagen, and (**f**) plasma-treated PLGA-collagen.

The DSC results for the untreated and plasma-treated PLGA, collagen, and PLGA-collagen samples are depicted in Figure 3a–f and compiled in Table 2. The main obstacle to generating a reliable DSC thermogram for collagen samples was the presence of entrapped HFP, as the crucible lid kept bursting at the 5 °C/min heating rate. This phenomenon was hypothesized to be a consequence of improper venting of the evaporating solvent that led to vapor entrapment and the foaming of the collagen samples (Figure S1, Supplementary Materials). To resolve this issue, a heating rate of 2.5 °C/min was applied for the collagen samples (Figure 3c,d).

According to the producer’s specifications, PLGA has a melting temperature of 157–164 °C. The argon plasma treatment appeared to have no effect on the peak melting temperature of PLGA (~155 °C in Figure 3a,b). As for collagen, thermal denaturation is an important parameter that is manifested as an endothermic peak in DSC thermograms. The temperature range for thermal denaturation depends on the collagen’s origin and is shifted to lower temperatures by reducing the heating rate [37]. The low-temperature transition events for collagen before and after plasma treatment (Figure 3c,d) were hard to interpret due to these thermograms being dominated by the evaporation of the entrapped HFP. Hence, the DSC transitions in Figure 3c,d at temperatures up to 150 °C were attributed to the combined effect of solvent evaporation and collagen denaturation. The thermal decomposition of collagen in Figure 3c,d was manifested by a sudden decline in the baseline, which has also been reported by others [37]. This interpretation is consistent with the corresponding TGA results (Figure 2c,d) showing pronounced mass loss at temperatures exceeding 150 °C, although the role of an entrapped solvent cannot be ruled out. As for PLGA-collagen, the samples showed a high level of fluctuation in their heat flow measurements, which could be attributed to the simultaneous phase transition of multiple components in the film. This included low-temperature endotherms with peak temperatures of 112.9 °C and 104.7 °C (Figure 3e,f, respectively), which could be attributed to solvent evaporation as well as to collagen denaturation [37]. These peak temperatures corresponded to the peak mass loss temperatures in the TGA thermograms (116.1 °C and 125.0 °C in Figure 2e,f, respectively). The lower heating rate used in DSC (vs. TGA) could explain the shift in these peak temperatures in Figure 3c,d. Comparing the peak melting temperatures in Figure 3e,f (157.9 °C vs. 159.2 °C) revealed that there was a negligible effect of the plasma treatment on PLGA-collagen films. Some major thermal events were present when there was a combination of PLGA and collagen in the formulation (Figure 3e,f), which corresponded to the onset of a pronounced mass loss in the TGA thermograms of PLGA-collagen films (Figure 2e,f). In the DSC measurements, these thermal events peaked at ~212 °C and ~223 °C for the untreated and plasma-treated PLGA-collagen, respectively (Figure 3e,f), which could be attributed to the thermal decomposition of collagen [37], although the role of entrapped HFP cannot be ruled out.

### 3.4. Contact Angle Measurements

**Figure 4:**
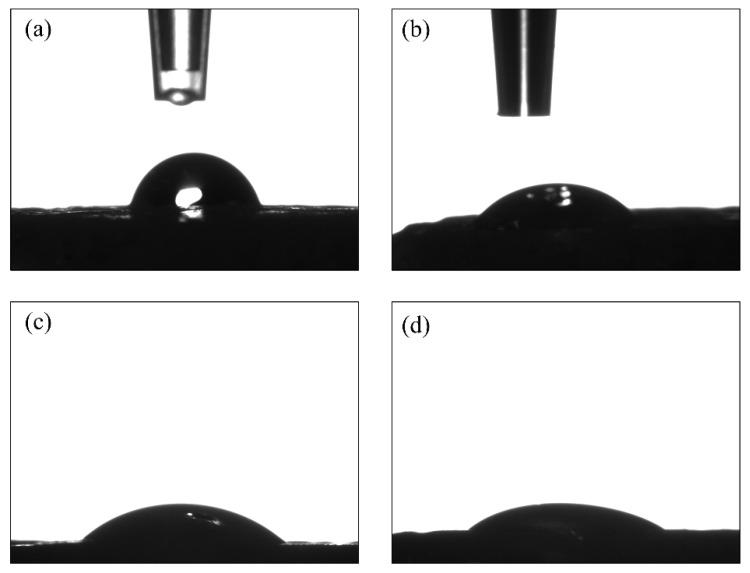
Contact angle measurements for the solvent-cast samples: untreated PLGA using (**a**) water and (**b**) diiodomethane droplets, and plasma-treated PLGA using (**c**) water and (**d**) diiodomethane droplets.

The contact angle measurements were performed in triplicate (n = 3) using water and diiodomethane (Table 3 and Figure 4). For the PLGA samples, the water contact angle was reduced from 70.0 ± 7.5° to 42.1 ± 0.5° after the plasma surface treatment (Table 3). A similar trend was seen with diiodomethane, albeit less pronounced, as the contact angle reduced from 53.4 ± 4.7° to 32.5 ± 0.9°. The standard deviations (SDs) were more notable for the untreated samples than for the plasma-treated ones. The hydrophobicity of PLGA decreased due to the effect of the argon plasma ablation of surface contaminants. The measurements of the collagen and PLGA-collagen samples were not conclusive because of the immediate diffusion of water into the collagen.

### 3.5. Critical Surface Tension (Surface Energy)

The contact angles of deionized water and diiodomethane were evaluated and extrapolated to θ = 0 (Zisman method). The critical surface tensions, which are the approximated surface energies of these samples, have been summarized in Table 4. The plasma-treated samples showed a higher level of variability in their critical surface tension. Hence, the difference between the plasma-treated and untreated groups was not statistically significant (*p* > 0.05).

### 3.6. Fourier-Transform Infrared Spectroscopy

**Figure 5:**
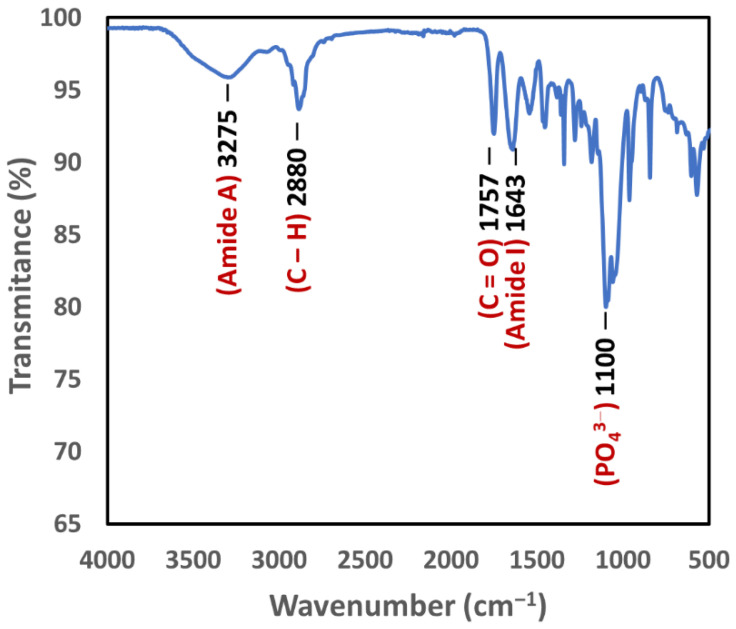
FTIR spectrum of a 3D bioplotted scaffold showing the presence of PLGA (carbonyl group) as well as collagen (amide A and amide I) and hydroxyapatite (PO_4_^3−^ ions).

The FTIR spectrum in Figure 5 shows the presence of PLGA in the 3D bioplotted scaffolds, as evidenced by a peak at 1757 cm^−1^ for carbonyl group (C=O) stretching [38]. The absorption peaks of the amide I bands have been reported to be between 1630 and 1657 cm^−1^ [39]. Hence, the presence of collagen in the scaffolds was based on the spectral absorption at 1642 cm^−1^ as well as the amide A peak related to N-H stretching (3275 cm^−1^). Hydroxyapatite could be identified via the absorption bands at 1105 cm^−1^ and 1035 cm^−1^, which were assigned to the phosphate ions (PO_4_^3−^) [40]. Furthermore, the absorption within the 2800–3000 cm^−1^ range (peaked at 2880 cm^−1^) was attributed to aliphatic C–H stretching [41].

### 3.7. Bicinchoninic Acid Assay

Based on the BCA assay results (Table 5), it was found that the plasma-treated scaffolds had a lower collagen release into PBS++ (0.288 ± 0.09 μg/μL), whereas the untreated scaffolds had a higher release of collagen (0.655 ± 0.10 μg/μL) into the environment, and the difference between the two groups was statistically significant (*p* < 0.05).

## 4. Discussion

It has been generally accepted that plasma surface modifications can improve the surface wettability [42,43,44,45] and cellular attachment [15,16,46,47,48,49,50] of polymeric scaffolds. A plasma surface treatment can be advantageous in supporting cell adhesion due to the added functionality on the cell–biomaterial interface in their early regeneration phase [46]. Once the cells are matured and ensconced in the extracellular matrix (ECM), they start producing their own differentiation and growth signals [47]. When a biomaterial is implanted in the body, it interacts with the host via its contact with salt ions and water molecules as well as with blood proteins [51]. These proteins may influence the eventual behavior of the recruited cells at the healing site [52]. Several studies have investigated the protein adsorption elicited by the plasma treatment of different classes of biomaterials and highlighted the potential biological benefits of plasma treatment [52,53,54].

This study aimed to enhance the hydrophilicity of PLGA/collagen-based biomaterials via an argon plasma surface treatment. We investigated the effect of argon plasma on several attributes of the synthetic/natural polymer systems (i.e., PLGA, collagen, and PLGA-collagen) prepared by solvent casting. Our study demonstrated that the water and diiodomethane contact angles on PLGA films were significantly reduced after the plasma treatment. These results are consistent with previously reported studies on an oxygen plasma treatment of poly(lactic acid) (PLA) scaffolds [42,55] and PLGA films [56]. The wettability of PLGA-based biomaterials has been reported to be a function of the lactide/glycolide ratio, and the wettability can be enhanced after plasma treatments [56].

The surface energy measurements using the Zisman method were not conclusive in this study. The Theta Lite Optical Tensiometer has a dual dispensing system that is intended to be used with water and diiodomethane. The measured water and diiodomethane contact angles were then used by OneAttension software to construct a Zisman plot based on the two measurements. Testing with additional liquids in future studies could help to quantify the effect of the plasma treatment on the surface energy of PLGA. Preparing solvent-cast films with a smoother surface topography could also help in reducing the fluctuations in the surface energy measurements. A high level of discrepancy has been reported between the values of the contact angles obtained by different investigators, which could be attributed to surface topographies as well as the humidities and diameters of the liquid drops used in the contact angle measurements [19].

The thermogravimetric characteristics of the PLGA-collagen films, analyzed via TGA measurements, predominantly resembled those of the PLGA films. This was despite the fact that PLGA and collagen were constituents of equivalent mass in the formulation. The thermal stability of PLGA can exceed 200 °C, as reported in other studies [57,58]. The thermal decomposition of PLGA remained within a narrow temperature range (322–346 °C) whether untreated or plasma-treated and with or without collagen in these formulations (Figure 2). Our results concur with a previous study on the thermal characterization of bulk PLGA [58], indicating that PLGA experienced primary weight loss in the range of 260–380 °C and negligible weight loss after 400 °C. The presence of HFP, which is a volatile solvent, had a pronounced effect on the TGA thermogram of PLGA. The onset of sample weight loss in Figure 2a marked the boiling temperature of HFP (~59 °C). Nevertheless, the evaporation of the HFP trapped inside the PLGA film appeared to take place in a broad temperature range and peaked at around 120°C. As for collagen, the untreated sample underwent a two-stage thermal event [59] with an initial degradation temperature of 280 °C and a secondary degradation temperature of 536 °C (Figure 2c). The argon plasma treatment appeared to have a negligible effect on the thermal degradation of collagen and PLGA–collagen at high temperatures. Nevertheless, the plasma-treated PLGA showed a slightly higher thermal degradation temperature (341 °C vs. 322 °C), although this might require further investigation if the high-temperature stability of PLGA would be of specific interest.

The TGA analyses of the samples were supplemented with DSC measurements in this study. Detecting the phase transitions or thermal events for which mass is invariant cannot be accomplished using TGA. Hence, coupling TGA with DSC can help with separating chemical changes from physical phenomena [60]. The argon plasma treatment appeared to have no effect on the peak melting temperature of PLGA in the DSC measurements. This was consistent with previous investigations of argon-plasma-treated biodegradable polyesters, indicating negligible effects on their crystallinity and melting temperature with a low plasma exposure time of 4–5 min [33,61]. DSC measurements are also well suited for analyzing thermal transitions in proteins, including the thermal stability and denaturation of collagen [62]. For example, DSC measurements have revealed a shift in collagen denaturation towards high temperature ranges after exposure to a low-temperature plasma jet generated in ambient air. This can be attributed to the ability of the plasma treatment to stabilize the collagen structure by stiffening its chains via a crosslinking action without altering its triple-helical structure [62].

Protein denaturation is an endothermic process, as it involves the dissociation of intramolecular bonds [63,64]. The temperature range for thermal denaturation in DSC measurements depends on the collagen’s origin [37], although DSC peaks tend to shift to lower temperatures when reducing the heating rate [37,65]. Hence, the DSC transition events for collagen at temperatures of up to 150 °C can be attributed to the combined effect of collagen denaturation and solvent evaporation (Figure 3c,d). This interpretation is consistent with the corresponding TGA results for collagen (Figure 2c,d), showing continuous mass loss over a wide temperature range due to solvent evaporation. Furthermore, the thermal decomposition of collagen was manifested by a sudden decline in the baseline at temperatures exceeding 150 °C (Figure 3c,d), which has also been reported in other studies [37]. Again, this was consistent with the corresponding TGA results (Figure 2c,d) showing a pronounced mass loss above 150 °C, which was also reported in other studies [37,66], although the role of entrapped HFP cannot be ruled out. Similarly, some major thermal events were present when there was a combination of PLGA and collagen in the formulation (Figure 3e,f), which corresponded to the onset of a pronounced mass loss in the TGA thermograms of the PLGA-collagen films (Figure 2e,f). In the DSC measurements, these thermal events peaked at ~212 °C and ~223 °C for the untreated and plasma-treated PLGA-collagen, respectively (Figure 3e,f), which might be attributed to the reported stiffening of collagen chains via plasma-induced crosslinking [62]. It is likely that the higher level of HFP entrapped in the collagen films prevented a similar observation in Figure 3c,d (See Figure S1, Supplementary Materials). It is worth emphasizing that the entrapped HFP was a major source of complexity in analyzing the TGA and DSC measurements. This study, as well as our previous work [20], has demonstrated the importance of taking this factor into consideration when solvent-processed biomaterials are used in research studies.

The PLGA-collagen formulations were loaded with nHA and PEG to enhance their osteoconductivity and hydrophilicity and were subsequently processed using a 3D Bioplotter. The FTIR spectrum of a 3D bioplotted scaffold showed the presence of PLGA (a band at 1757 cm^−1^ for the carboxyl group) [38], collagen (bands at 1642 cm^−1^ and 3275 cm^−1^ for amides) [39], and hydroxyapatite (a band at ~1100 cm^−1^ for PO_4_^3−^ ) [40]. Furthermore, the absorption within the 2800–3000 cm^−1^ range (peaked at 2880 cm^−1^) was attributed to aliphatic C-H stretching [41]. Previous FTIR studies have shown that the argon plasma treatment of collagen reduces the intensity of the characteristic amide bands while increasing the intensity of a peak representing C-O-C linkage, which appears to be a consequence of plasma-induced reactions affecting the chemical composition of the surface [41,67]. This has been further confirmed via a surface analysis using X-ray photoelectron spectroscopy (XPS), showing a pronounced increase in the oxygen content of the surface upon argon plasma treatment and leading to an increase in the O/C ratio due to surface oxidization [41].

The BCA analysis revealed that the plasma-treated 3D scaffolds had a lower collagen release into the PBS, and the difference compared to the untreated scaffolds was statistically significant. Hence, the influence of argon plasma surface modification appeared to include the structural crosslinking of collagen in these scaffolds [32].

## 5. Conclusions

This study presented the surface modification of PLGA, collagen, and PLGA-collagen solvent-cast films via an argon plasma treatment. These biomaterials underwent liquid contact angle analyses as well as TGA and DSC measurements. The water contact angle on PLGA films was significantly reduced after the plasma treatment (*p* < 0.05). The TGA analysis indicated that the thermal decomposition of PLGA remained within a narrow temperature range for both the untreated and plasma-treated formulations and that the argon plasma treatment appeared to have a negligible effect on the thermal degradation of collagen and PLGA–collagen at high temperatures. The DSC transition events for collagen at temperatures of up to 150 °C were attributed to the combined effect of collagen denaturation and solvent evaporation. Some major thermal events were present in the DSC thermograms when there was a combination of PLGA and collagen in the formulation. The thermal transitions peaked at ~212 °C and ~223 °C for the untreated and plasma-treated PLGA-collagen, respectively, corresponding to the onset of a pronounced mass loss in the TGA thermograms of PLGA-collagen. This might be attributed to the reported stiffening of collagen chains via plasma-induced crosslinking, although further investigation is needed to draw a conclusion. The PLGA-collagen formulations were also loaded with nHA and PEG and processed using a 3D Bioplotter. The influence of the argon plasma surface modification was found to be the structural crosslinking of the collagen in these scaffolds, as evidenced by the BCA analysis. These biomimetic composite formulations will be further investigated in our future in vivo trials. Overall, this study also demonstrated the importance of taking the entrapped solvent into consideration when solvent-processed biomaterials are used in research studies.

## Figures and Tables

**Table 1:** Compositions of the samples prepared by solvent casting.

Sample #	Sample Name	Argon Plasma	PLGA Mass (g)	Collagen Mass (g)	HFP Volume (mL)
1	Untreated PLGA	No	0.30	0	0.51
2	Plasma-treated PLGA	Yes	0.30	0	0.51
3	Untreated collagen	No	0	0.20	1.50
4	Plasma-treated collagen	Yes	0	0.20	1.50
5	Untreated PLGA–collagen	No	0.20	0.20	1.84
6	Plasma-treated PLGA–collagen	Yes	0.20	0.20	1.84

**Table 2:** Thermodynamic parameters for the DSC heating ramp (endothermic phenomena).

Sample #	Sample Name	T_peak_ (°C)	ΔH (J/g)
1	Untreated PLGA	154.7	39.2
2	Plasma-treated PLGA	155.2	33.9
3	Untreated collagen	-	-
4	Plasma-treated collagen	-	-
5	Untreated PLGA–collagen	112.9 157.9 211.8	27.0 - 8.40
6	Plasma-treated PLGA–collagen	104.7 159.2 223.1	28.9 - 24.7

(-) The parameters skewed by HFP evaporation have been omitted from this table.

**Table 3:** Contact angle measurements of the solvent-cast PLGA samples.

Sample #	Liquid	Measurement 1 (°)	Measurement 2 (°)	Measurement 3 (°)	θ (°)
Untreated PLGA	Water	73.7	76.8	59.6	70.0 ± 7.5
	Diiodomethane	55.1	58.1	47.0	53.4 ± 4.7
Plasma-treated PLGA	Water	42.8	42.0	41.6	42.1 ± 0.5
	Diiodomethane	31.1	33.0	33.3	32.5 ± 0.9

**Table 4:** Surface energy analysis of the solvent-cast PLGA samples via the Zisman method.

Sample #	Sample Name	Measurement 1 γ (mN/m)	Measurement 2 γ (mN/m)	Measurement 3 γ (mN/m)	Mean ± SD γ (mN/m)
1	Untreated PLGA	18.2	16.0	11.0	15.1 ± 3.0
2	Plasma-treated PLGA	25.0	13.7	9.7	16.1 ± 6.5

**Table 5:** Concentration of the collagen released into PBS++ (3D bioplotted scaffolds).

Sample #	Sample Name	Measurement 1 (μg/μL)	Measurement 2 (μg/μL)	Measurement 3 (μg/μL)	Mean ± SD (μg/μL)
1	Untreated scaffold	0.516	0.723	0.725	0.655 ± 0.10
2	Plasma-treated scaffold	0.237	0.207	0.420	0.288 ± 0.09

## Data Availability

Not applicable.

## Supplementary Materials

The following supporting information can be downloaded at: https://www.mdpi.com/article/10.3390/biomimetics7040218/s1, Figure S1: Collagen/HFP foam generated at a 10 °C/min DSC heating rate. The image shows the rupture of the DSC pan following the foaming process.
