# Supplementary material for: The Effect of Argon Plasma Surface Treatment on Poly(lactic-co-glycolic acid)/Collagen-Based Biomaterials for Bone Tissue Engineering
Source: Biomimetics (Basel). 2022 Nov 29;7(4):218. doi: 10.3390/biomimetics7040218 (PMC9776356; doi:10.3390/biomimetics7040218)
Supplement: Supplementary file 1 [file biomimetics-07-00218-s001.zip › biomimetics-2029782-supplementary.pdf]

biomimetics

Supplementary Materials

Phat T. Vu [1], Jackson P. Conroy [1] and Amy M. Yousefi [1,*]

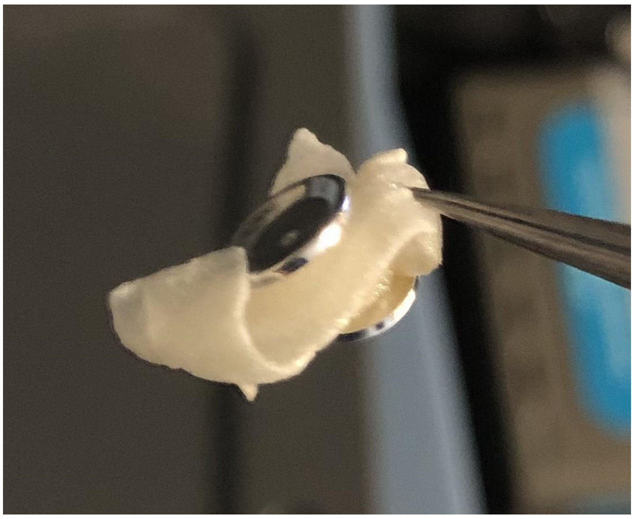

Figure S1. Collagen/HFP foam generated at 10°C/min DSC heating rate. The image shows the rupture of the DSC pan following the foaming process.
